# Immunopathology of Atherosclerosis and Related Diseases: Focus on Molecular Biology

**DOI:** 10.3390/ijms22084080

**Published:** 2021-04-15

**Authors:** Evgeny E. Bezsonov, Igor A. Sobenin, Alexander N. Orekhov

**Affiliations:** 1 Laboratory of Angiopathology, Institute of General Pathology and Pathophysiology, 8 Baltiiskaya Street, 125315 Moscow, Russia; igor.sobenin@gmail.com; 2 Laboratory of Cellular and Molecular Pathology of Cardiovascular System, Institute of Human Morphology, 3 Tsyurupa Street, 117418 Moscow, Russia; 3 Department of Biology and General Genetics, I.M. Sechenov First Moscow State Medical University (Sechenov University), 2-4 Bol’shaya Pirogovskaya Ulitsa, 119435 Moscow, Russia; 4 Laboratory of Medical Genetics, National Medical Research Center of Cardiology, 15a 3rd Cherepkovskaya Street, 121552 Moscow, Russia

In this Special Issue of the *International Journal of Molecular Sciences*, we include insightful reviews and research papers on the subject “Immunopathology of Atherosclerosis and Related Diseases: Focus on Molecular Biology”. We would like to share first some background about this topic.

Chronic inflammation is a pathogenic mechanism of many diseases causing up to 80% of morbidity and mortality in developed countries. Despite many discoveries made in the field of innate immunity, the exact causes of uncontrolled inflammation are still to be discovered [1,2,3,4]. Atherosclerosis represents a good example of a disease associated with chronic inflammation at all stages of pathogenesis development, starting from an early to an advanced one. When a local lesion happens in a muscular-elastic artery, an atherosclerotic plaque is formed. The lesion grows up slowly (for many years), reducing the blood flow in the artery with a possibility of a complete blockage due to thromboembolism. The most dangerous lesions for a patient’s life typically happen in the aorta, coronary arteries, brain arteries, renal arteries, and arteries of lower limbs. Three phases of inflammation were found to exist in atherosclerotic lesions on a tissue level: an infiltration, reparation, and scar phase. The infiltrative stage of inflammation (swelling) is related to the initial atherosclerotic lesion (lipid infiltration). Advanced lesions (fatty streaks and fibrolipid plaques) are characterized by the formation of a lipid core. The reparative phase consists of cell proliferation and synthesis of connective tissue and occurs together with lipidosis. In the next step, a scar (a fibrous cap) is formed in the atherosclerotic lesion.

Innate immune cells and their mediators are critical for the initiation and development of atherosclerotic inflammation. Several innate immune cell types were found in the arterial walls in the animal models and patients [1].

Innate immunity is actively involved in the development of atherosclerosis since the number of immune cells was significantly increased in atherosclerotic lesions in comparison with healthy regions [2].

The evolutionary most conservative system of host defense against pathogens is innate immunity. Pathogen-associated molecular patterns (PAMPs) and damage-associated molecular patterns (DAMPs) serve as main inflammatory initiators. However, modified low-density lipoproteins (LDL), while by their nature being neither PAMPs nor DAMPs, are still able to induce sterile (or noninfectious) inflammation in the case of atherosclerosis. In every cell, there is a potential source of DAMPs capable of contributing to the stimulation of innate immunity called mitochondria. Mitochondria are eukaryotic organelles originating from bacteria and carrying their own genome and some other bacterial traits such as the presence of prokaryotic phospholipid cardiolipin in the mitochondrial inner membrane. There is a certain similarity between bacteria and mitochondria and the ability of defective mitochondria to induce immune response was shown when cells are damaged mitochondrial DAMPs get released.

A group of protein complexes, inflammasomes, are involved in the response to PAMPs and DAMPs stimuli, which results in the production of major pro-inflammatory cytokines, interleukin-1β and IL-18, and cell death. Mitochondria are believed to play an important role in the initiation and regulation of NLRP3 inflammasomes [5]. NLRP3 activators cause the release of cardiolipin (required for activation of inflammasomes [6]), mitochondrial DNA, and mitochondrial reactive oxygen species. When activated, inflammasomes cause Caspase-1 dependent mitochondrial damage and block of mitophagy [7], which stimulates an immune response (it is especially important in case of bacterial infections and regulation of immunity and metabolism). Mitophagy is a part of a quality control system recognizing and degrading damaged mitochondria through lysosomes [8], and defects in this system result in an accumulation of dysfunctional mitochondria and cytokine-induced inflammation [9].

It is known that mitochondrial DNA (mtDNA) mutations are associated with many different diseases including cancer, pulmonary fibrosis, asthma, cystic fibrosis, pulmonary hypertension, autoimmune rheumatic diseases, and aging. A correlation between certain mtDNA mutations and atherosclerosis was found using leukocytes of atherosclerotic patients [10,11,12]. Cybrid cell lines carrying different variants of the mitochondrial genome from atherosclerotic patients allowed to identify: 1. mutations of mtDNA uncoupling oxidative phosphorylation (del562G, m.1555A>G, m.14459G>A, and m.14846G>A); 2. mutations of mtDNA sustaining ATP synthase activity (m.3256C>T, m.12315G>A, and m.13513G>A); 3. mutation associated with a higher basal rate of oxygen consumption (m.14459G>A) [13].

The presence of atherosclerosis-associated mitochondrial mutations correlated with pro-inflammatory activation of monocytes isolated from the blood of patients with asymptomatic atherosclerosis. The level of monocyte activation correlated with the presence of two homoplasmic mutations m.1811A>G and m.9477G>A and three heteroplasmic mutations m.14459G>A, m.1555A>G, and m.12315G>A [14].

mtDNA mutants associated with the increased mitophagy (was tested by LAMP gene expression in cybrids carrying atherosclerosis-associated mtDNA mutations) were identified: m.3336T>C, m.3256C>T, and m.5178C>A [13].

Based on all available data mentioned above, the hypothesis explaining the causes of atherogenicity was proposed [15]. The lipid accumulation in the arterial wall cells of atherosclerotic patients gets induced by the atherogenic modified LDL circulating in the blood and penetrating the cells by nonspecific phagocytosis in the form of self-associates [16,17]. The pro-inflammatory response in macrophages in the form of the secretion of inflammatory cytokines gets activated upon the stimulation of phagocytosis by modified LDL associates [18]. An increased accumulation of intracellular lipids is happening upon the cytokine secretion [19], which, in normal situations, stops, aptly thus preventing an excessive lipid uptake. However, the presence of mtDNA mutations in macrophages can prevent the arrest of pro-inflammatory response or even increase it upon repeated stimulation. Defective mitophagy could be the reason for mitochondrial dysfunction. An atherosclerotic lesion appears due to local inflammation in the arterial wall, which becomes chronic and goes along with an uncontrolled lipid buildup. An additional possible pro-inflammatory factor of pathogenicity is that damaged or dysfunctional mitochondria may be recognized by cells as pathogens and trigger the immune response [20].

Additional conclusions could be made using the proposed hypothesis: atherosclerosis induction could be based on two recognition errors happening in cells of the arterial wall. The first error is related to the recognition of self-associates of modified LDL by the cell as pathogens with subsequent phagocytosis, inflammatory response, and intracellular lipids accumulation, which triggers atherogenesis at a cellular level. The second error is related to defective mitophagy caused by mitochondrial mutations. Defective mitophagy, in turn, causes disturbed innate immunity response, in which a continuous inflammatory signal is induced, leading to the chronification of inflammation.

Thus, the impaired innate immune response leads to the chronicity of inflammation in any pathology associated with inflammation. The model of atherosclerosis development due to chronic inflammation induced by modified LDL and impaired mitophagy is shown in Figure 1.

In this Special Issue, several important topics were discussed in published articles and reviews.

Casas with coauthors [21] found that chronic kidney disease (CKD) patients show an increased risk of Cardiac Surgery-Associated Acute Kidney Injury and mortality after cardiovascular surgery, associated with the expansion of the CD14^++^CD16^+^ subset of pro-inflammatory monocytes and with IL1β expression. They proposed that inflammation associated with CKD may contribute to atherosclerosis pathogenesis.

Zhang with coauthors [22] employed the CIRCULATING CELLS study cohort to classify cardiovascular disease patients and healthy individuals in relation to their expression of neuroimmune guidance cues in circulating monocytes. They found that the linear discriminant analysis, Naïve Bayesian model, and stochastic gradient boost model yielded perfect or near-perfect sensibility and specificity, and revealed that expression levels of the neuroimmune guidance cues SEMA6B, SEMA6D, and EPHA2 in circulating monocytes were of predictive values for cardiovascular disease outcome.

Terasaki with coauthors [23] examined whether a DPP-4 inhibitor, teneligliptin, could suppress oxidized low-density lipoprotein (ox-LDL) uptake, foam cell formation, CD36, and acyl-coenzyme A: cholesterol acyltransferase-1 (ACAT-1) gene expression of macrophages isolated from streptozotocin-induced type 1 diabetes (T1D) mice and T1D patients, as well as advanced glycation end product (AGE)-exposed mouse peritoneal macrophages and THP-1 cells. The findings of the authors suggest that teneligliptin may inhibit foam cell formation of macrophages in T1D via suppression of CD36 and ACAT-1 gene expression partly by attenuating the harmful effects of AGEs.

Sotokawauchi with coauthors [24] found that GLAP is one of the structurally distinct glycer-AGEs, which may mediate oxidative stress and inflammatory reactions in glycer-AGE-exposed tubular cells. Blockade of the interaction of GLAP-RAGE by GLAP-aptamer may be a therapeutic target for proximal tubulopathy in diabetic nephropathy.

Yeh with coauthors [25] in their review presented current knowledge regarding the roles of microbiota in contributing to atherosclerotic pathogenesis and highlighted translational perspectives by discussing the mutual interplay between microbiota and the immune system on atherogenesis.

Puig with coauthors [26] in their review discussed the concept of vulnerable carotid plaque, and collected existing information about putative circulating biomarkers, being particularly focused on lipid-related and inflammatory molecules.

Kandarakov and Belyavsky [27] in their review discussed the phenomenon of clonal hematopoiesis, the most important genes involved in it, its impact on cardiovascular diseases, and relevant aspects of hematopoietic stem cell biology.

Okhota with coauthors [28] in their review discussed von Willebrand factor involvement in complications of cardiovascular diseases and possible diagnostic and treatment approaches.

Rychter with coauthors [29] in their review summarized the current knowledge about RBP4 and its association with essential aspects of cardiovascular disease—lipid profile, intima-media thickness, atherosclerotic process, and diet. They also discussed the RBP4 gene polymorphisms essential from a cardiovascular perspective.

Mori with coauthors [30] in their review summarized the cardiovascular effects of the glucose-dependent insulinotropic polypeptide (GIP) and GIP receptor agonists (GIPRAs) in cell culture systems, animal models, and humans. Recently, pharmacological doses of GIPRAs have been found to exert anti-obesity effects in animal models. These observations suggest that combination therapy of glucagon-like peptide-1 and GIP receptor may induce superior metabolic and anti-diabetic effects compared with each agonist individually.

In conclusion, we would like to express our appreciation of high-quality papers submitted to the Special Issue by the authors. Despite the great progress made in the field of the immunopathology of atherosclerosis and related diseases in this Issue and in other publications still, there are white spots that need to be covered in future research.

## Figures and Tables

**Figure 1:**
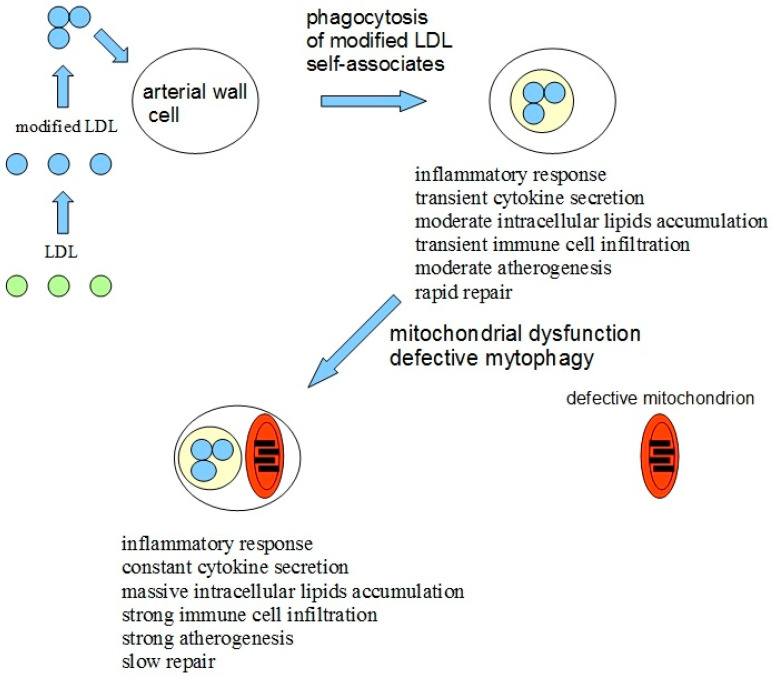
The model of atherosclerosis development due to chronic inflammation induced by a combination of recognition errors of modified low-density lipoproteins (LDLs) as pathogens and impaired mitophagy. Modified LDLs form self-associates and are identified by arterial cells as pathogens. After phagocytosis of LDL associates, a moderate inflammatory response is activated, leading to moderate intracellular lipid accumulation and moderate atherogenesis and the damage can still be repaired. However, in case of impaired mitophagy due to mitochondrial mutations, the inflammatory response, once being activated by modified LDL, does not stop and the inflammation becomes uncontrollable, leading to massive intracellular lipids accumulation, infiltration of immune cells, and strong atherogenesis. Modified and adapted from [15].
